# Notes and News

**Published:** 1900-09-29

**Authors:** 


### Notes and News.

Rather a serious outbreak of diphtheria has appeared in the village of Pill, Somerset. There are also a number of cases in Leicester.

Liverpool, West Derby, and Toxteth Park parochial authorities have decided to establish a hospital at Heswell, at a cost which is limited to £5,700.

It is announced that the Prince and Princess of Wales will visit Belfast next year, and lay the foundation-stone of the new Royal Victoria Hospital.

It is expected that about 60 unions and parishes will be represented at the Conference of Poor-Law Administrators to be held in October at St. Martin's Town Hall.

The Royal Alexandra Hospital for Children at Rhyl, of which the Princess of Wales laid the foundation-stone, has been opened during the present month. It provides for 160 inmates. The hospital has an uninterrupted sea frontage. The late Duke of Westminster contributed £10,000 towards the building.

The severe criticism which Mr. Burdett-Coutts has hurled against the military hospitals in South Africa has not found an echo in Canada. Dr. Ryerson, the Canadian Red Cross Commissioner, has returned to his country after completing his labours, and speaks most highly of his experience, as do the members of the Canadian contingent who passed through the hospitals. Dr. Ryerson testifies to the excellent manner in which the hospitals were conducted, in spite of the great difficulties the staff had to contend with.

University College Hospital, which has been closed since September 1st, was re-opened for the reception of both in-patients and out-patients on the 24th inst. In 1898 the Prince of Wales laid the foundation-stone of a new building which Sir J. Blundell Maple, Bart., had generously undertaken to erect at a cost of over £120,000. The first wing and the central block are now ready for occupation, and were informally opened on the 24th inst. The plan in its entirety will be in the form of a diagonal cross. Special attention has been given to the requirements of modern medicine and surgery, and each ward will be completely isolated, and be connected with the rest of the structure by bridges.

The late Mr. J. Jefferies has bequeathed £1,000 to the East Suffolk and Ipswich Hospital to endow a bed in the new Victoria wing.

Sir William MacCormac will distribute prizes to the medical students of St. Thomas's Hospital in the Governors' Hall on Tuesday, October 2nd, at three p.m.

Mr. H. Stansfield Collier, F.R.C.S., will deliver the opening address at St. Mary's Hospital Medical School at the hospital, on Monday, October 1st, at half-past three p.m.

Sir William MacGregor, K.C.M.G., C.B., M.D., will deliver the opening address of the autumn session of the London School of Tropical Medicine on Wednesday, October 3rd, at half-past eight p.m., at the school.

At the half-yearly board of the governors of the Bristol Royal Infirmary, held on Tuesday last, it was unanimously resolved, on the motion of the Dean of Bristol (chairman), seconded by Dr. Shingleton Smith, that the warmest thanks of the governors be accorded to Mr. Richardson Cross (who had resigned the office of honorary ophthalmic surgeon) for the very valuable services he had rendered to the institution throughout the last 22 years, and that he be appointed honorary and consulting ophthalmic surgeon. At a subsequent meeting of the Committee of Election, Dr. Alexander Ogilvy, B.A., B.S. and M.D. (Dublin Univ.), and F.R.C.S.I., was unanimously elected honorary ophthalmic surgeon in the place of Mr. F. Richardson Cross, M.B., F.R.C.S., resigned.

The Bradford Guardians have been considering the question of providing a consumption sanatorium for its pauper population. Practically a site has been selected, though the scheme cannot be carried out until the Local Government Board has given its sanction. There is no precedent for the establishment of a sanatorium by a single Board of Guardians, but the Bradford Board consider the requirements of at least 40 patients warrant the scheme. A very large number of phthisical patients pass through the Union Infirmary, and it is held that a larger number still would present themselves in the earlier stages of the disease if they could secure treatment in a sanatorium. The site favoured by the Board, after careful expert opinion having been obtained, is at Eastby, near Embsay. It is very sheltered, has a good water supply, and is near a railway station.

The Hertford British Hospital in Paris was, on the death of the late Lady Wallace, transferred to Her Majesty's Government, who have appointed a committee to manage the hospital, consisting of the Hon. Michael Herbert, C.B., Mr. Austin Lee, C.B., Mr. A. Percy Inglis (Her Majesty's Consul-General), Mr. John Pilter, and Mr. Alfred Coleman. Her Majesty's Office of Works recently instructed Professor Corfield, Inspector of Buildings for the Department, to investigate the sanitary arrangements of the hospital, and he has reported that in his opinion the hospital, which had been closed for temporary repairs, cleaning, &c., should not be reopened until extensive alterations have been made in the present sanitary arrangements. Meanwhile, in order to obviate any inconvenience which may result from the non-admission of urgent cases among the British poor resident in Paris, instructions have been given to the resident medical officer to take immediate steps to obtain admission to a suitable French hospital for any such applications.

